# Supplementary material for: The influence of receiving real-time visual feedback on breathing during treadmill running to exhaustion
Source: PLoS One. 2022 Jul 7;17(7):e0270335. doi: 10.1371/journal.pone.0270335 (PMC9262203; doi:10.1371/journal.pone.0270335)
Supplement: Attachment — Submitted filename: Response to Reviewers.docx [file pone.0270335.s001.docx]

**Rebuttal Letter**

We greatly appreciate the comments and points raised on our manuscript after review. Our responses are provided below, and the marked-up copy of the manuscript shows all the mentioned changes in red lettering.

**Journal Requirements**

**1. Please ensure that your manuscript meets PLOS ONE's style requirements, including those for file naming.**

Abbreviations have been removed from the author affiliations and the initials of the corresponding author have been added in parentheses after the email address. All section headings have been updated to follow sentence structure. All file names match the requirements and all figures have been uploaded to the PACE digital diagnostic tool and confirmed that they meet PLOS requirements.

**2. We note that you have stated that you will provide repository information for your data at acceptance. Should your manuscript be accepted for publication, we will hold it until you provide the relevant accession numbers or DOIs necessary to access your data. If you wish to make changes to your Data Availability statement, please describe these changes in your cover letter and we will update your Data Availability statement to reflect the information you provide.**

We do not wish to make changes to our data availability statement. All data will be available in a repository upon acceptance and the relevant accession numbers or DOIs to access the data will be provided at that time.

**3. Please include your full ethics statement in the ‘Methods’ section of your manuscript file. In your statement, please include the full name of the IRB or ethics committee who approved or waived your study, as well as whether or not you obtained informed written or verbal consent. If consent was waived for your study, please include this information in your statement as well.**

The approval number of our study has been added in line 87. The full name of the IRB committee as well as the statement that written informed consent was provided by participants prior to beginning the study is provided in lines 86-88.

**Additional Editor Comments**

**There are some concerns regarding the detail provided in the methods for using the RIP sensors. Therefore in the methods can you please provide:**

**1) Details regarding raw signal processing and breath onset detection**

**2) A raw data trace of the signal.**

Raw data traces and the processing of data to calculate phase angle are now provided in the newly added Fig 5. The details regarding this raw signal processing and breath onset detection have been added in lines 174-192. Limitations pertaining to the processing of RIP data during running are addressed in lines 314-319.
