# Supplementary material for: The influence of receiving real-time visual feedback on breathing during treadmill running to exhaustion
Source: PLoS One. 2022 Jul 7;17(7):e0270335. doi: 10.1371/journal.pone.0270335 (PMC9262203; doi:10.1371/journal.pone.0270335)
Supplement: Attachment — Submitted filename: Response to Reviewers.docx [file pone.0270335.s002.docx]

**Rebuttal Letter**

We greatly appreciate the comments from the reviewers, our response to each comment is provided in the table below. Any mention of changes to the manuscript are shown in the marked-up copy in red lettering.

**Reviewer Comments to the Author**

| **Reviewer Comment** | **Our Response** | **Changes to Manuscript** |
| --- | --- | --- |
| Line 31 “breathing synchronicity” – better to use one of the accepted terms “thoraco-abdominal coordination” or “thoraco-lumbar synchrony” | The term “thoraco-abdominal coordination” is now used. | “Breathing synchronicity” replaced with “thoraco-abdominal coordination” in line 31. |
| Lines 41-42 is this reference correct? | The reference was made to the proper study but the citation was missing information. The citation has been updated. | Added information to complete the citation of reference 1 in line 353. |
| Lines 44-46 not sure this connects the previous and next sentences… can you provide info re: how breathing provides o2 and clears co2? | We have provided further information on oxygen and carbon dioxide, which also helps connect the mentioned sentences. | Lines 44-45 expand on oxygen and carbon dioxide during breathing. |
| Lines 49-50 this is a brief, bold statement supported by much theoretical knowledge but scarce experimental evidence. Citations 9-11 do not refer to T-L synchrony during exercise. Can you expand the concept of T-L synchrony independently before introducing RIP? | It is correct that citations 9-11 do not refer to breathing during exercise, but they do still show the value of RIP and the importance of thoraco-abdominal coordination. Expansion on the concept of thoraco-abdominal coordination has been added. | Expanded on the concept of thoraco-abdominal synchrony in lines 51-53. |
| Lines 53-57 I suggest you check some updated references on RIP validity as there appear to be advances in this space in the last 10y. Specifically there are many recent studies on another commercial RIP device (Hexoskin) | Thank you for bringing this to our attention. We have added a citation to your suggested study by Harbour et al. to further emphasize that RIP has been shown to be reliable during exercise. | Added reference in line 58. A citation for Harbour et al. has been added to the references list. |
| Lines 54-57 there are several studies examining the effect of breathing pattern interventions upon running | Thank you for providing these sources, although it appears that Bahensky et al. did not have participants run to assess the breathing intervention. Regardless, these articles are important to highlight the fact that studies on altering breathing pattern during exercise have been done before. | Added a statement and references to these breathing pattern intervention studies in lines 58-59. Citations for Bahensky et al. and Matsumoto et al. have been added to the references list. |
| Lines 61-62 please populate this with normal values during exercise (Ref 16?). | The average phase angle found by Clarenbach et al. has been added. | Lines 64-66 have been edited to include an average phase angle during exercise. |
| Lines 72-76 can you separate and clarify these points? Specifically, “breathing in healthy individuals is typically not optimally efficient” is a large claim likely difficult to substantiate with research. Maybe you mean to specify that healthy exercisers present with suboptimal T-L synchrony values and that improvement might also affect performance? | These lines are in place to build on the previous points regarding average phase angle, whether at rest or exercising. It was assumed that participants were not breathing as efficiently as they possibly could be (i.e., a phase angle greater than 0 degrees), ultimately leading to the purpose of the studying being to try to improve this breathing efficiency and see if in turn time to exhaustion increased. | None. |
| Lines 80-86 can be easily be combined and made more concise. | The paragraph containing this information has been reorganized to be more concise. | Restructured lines 85-88. |
| Lines 176-187 these lines all describe breath/flow reversal detection; can you explicitly label as such? Furthermore, if you have TV data, why not present this in results? Just a suggestion if there might be interesting comparisons. | The term “breath reversal detection” has been added. Tidal volume data are not included due to the focus of the study being on thoraco-abdominal coordination. We did not feel that tidal volume data would significantly contribute to the results or discussion. | “Breath reversal detection” added in line 190. |
| Lines 190-192 averages are good; do you have any other time-series analysis metrics that might show different aspects of breathing pattern? Propose variability of phase angle (as SD or CV) or slope (change from beginning to end). If you do not have these, include this caveat in your discussion. | We do not have time-series metrics to add primarily due to a lack of a significant difference being observed between the two testing days. A comment on this has been added to the limitations section. | A comment regarding time-series analysis in future studies has been added in lines 317-318. |
| Lines 252-255 is this really the most similar study for comparison? Suggest to compare instead with another breathing strategy study using time to exhaustion | The treadmill protocol used in this study was based off of Fraga’s work. Given that both populations underwent similar protocols to exhaustion, this was considered the most similar study to our own even though it did not focus on breathing. | None. |
| Lines 255-256 have you considered the unique constraints on breathing that running confers versus cycling and other sports? | There are certainly a wide range of breathing constraints a person will experience based on the physical activity that is being performed. We ultimately chose running due to its popularity and the potential impact these results could have on the running community. | None. |
| Lines 258-259 do you have any justification for this statement that experienced runners have a more efficient breathing pattern? | It was an assumption that more experienced runners were able to exhibit better breathing patterns. We also theorized that experienced runners were more likely to have already learned some type of breathing technique than a less experienced runner. | Lines 263-265 have been restructured to state this assumption. |
| Lines 260-266 does not strengthen this discussion for me. If you wish to retain, please expand upon why this protocol selection is important: is it better simply for intra-subject equivalence? Is it superior for eliciting respiratory limitations? | Given that we had a wide range of runners we had to choose a protocol that could fit our population, which ultimately was not a constant-pace protocol (reasoning explained in the methods section). If we had more time to tailor the protocol to each participant at a specific constant pace for them, it is possible that we could have observed a greater difference between testing days as the other mentioned studies with similar research objectives did. | Added a statement to the end of this paragraph to emphasize why this protocol was chosen in lines 272-274. |
| Lines 279-281 can you put this in context with the results of Bernardi, 2017 as mentioned above? What does T-L synchrony have to do with insp/exp airflow and the work of breathing? | Newly added information in response to a previous comment in lines 51-53 helps address this. Similar information has been added here to re-emphasize this. | Re-added information in lines 289-290 to emphasize the importance of thoraco-abdominal coordination. |
| Lines 281-284 do you have any idea what amount of time is needed to optimize this familiarization? There should be some contextual comparison in biofeedback studies and some of the previous references in learning breathing techniques. | We feel that a pilot study with this visual feedback system could be done to gauge how long it takes participants to fully feel comfortable with the system. Tracking phase angle with visual feedback over time can ensure that improvements are being made and find the point in time where people exhibit the most improvement. Given that this visual feedback system has not been used in any other studies before, it’s difficult to confidently estimate how long this would take without another study being done. | Added a statement to the limitations that more work is needed to fully understand the amount of time that a participant needs to become fully comfortable with the visual feedback system in lines 314-316. |
| Lines 285-288 clunky; reword | These lines have been reworded. | Reworded lines 294-296. |
| Can you add some comments to the discussion re: field vs. laboratory running? The implication here is that this breath retraining might have long-term effects on breathing during running. Can you also make a comment on feedback learning/self-awareness of T-L synchrony if expected to carry into field running? | We feel that this goes beyond the scope of this study given the results. We weren’t able to reliably induce a change in thoraco-abdominal coordination in the laboratory, so it seems premature to comment on transfer of the skill to other situations. Had significant differences been observed between testing days, the theory of these results translating from lab running to field running could be commented on. | None. |
| Lines 297-302 a large limitation I see is missing subjective feedback such as dyspnea, perceived efficacy/difficulty (to breathing instructions), and cognitive load. Please comment. | Given that participants were undergoing the same protocol and ideally going to their exhaustion point on each day, we did not feel it was necessary to collect subjective feedback. While we did not collect feedback in a structured way, participants were instructed to report any confusion in instructions or shortness of breath while running, and ultimately no participants did. | Added notes on the unstructured feedback that was collected during the study in lines 309-313. |
| Line 300-301 first part of sentence says nothing; how about “manual breathing re-education methods” or the like? | The term “manual breathing re-education methods” has been added. | Added “manual breathing re-education methods” in lines 313-314. |
| Lines 303-306 also consider the limitations of condition randomization – the order of verbal vs visual cues might have affected the instruction adherence of the next session. | There was not a significant difference observed in time to exhaustion for the order of testing visits, which would suggest that the cues on day 1 would not affect day 2. | Expanded on this point in lines 322-324. |
| Can you sharpen figure 5? Not only it is blurry, but also unclear. Specifically, I am guessing that there should not be an arrow between the summed tidal volume and phase angle graphs. | The plots in Fig 5 have been enlarged to make them easier to read. The arrows are simply included to help continue the flow from the top of the figure to the bottom. | Enlarged plots in Fig 5 to sharpen the image. |
