# Supplementary material for: The influence of receiving real-time visual feedback on breathing during treadmill running to exhaustion
Source: PLoS One. 2022 Jul 7;17(7):e0270335. doi: 10.1371/journal.pone.0270335 (PMC9262203; doi:10.1371/journal.pone.0270335)
Supplement: Attachment — Submitted filename: Response to Reviewers.docx [file pone.0270335.s004.docx]

**Rebuttal Letter**

We appreciate the additional comments from the reviewers, our response to each comment is provided in the table below. Any mention of changes to the manuscript are shown in the marked-up copy in red lettering.

**Reviewer Comments to the Author**

| **Reviewer Comment** | **Our Response** | **Changes to Manuscript** |
| --- | --- | --- |
| Lines 52-53: while the second clause is welcome detail, the third does not fully substantiate the basis for this study to use T-L coordination. What does T-L coordination have to do with oxygen perfusion into the bloodstream? I don’t see a clear connection here either theoretically or experimentally based on the references. Maybe the real justification (especially in exercise) has to do with respiratory muscle fatigue? Or perhaps expiratory flow limitation? Please revise (also 289-290). | This is a crucial aspect of this study and we thank the reviewer for pointing it out. The original 3 points have been expanded on by going into details on T-L coordination and its benefits when applied, as well as breathing’s role in exertion and oxygen exchange during exercise. While the combination of these points is theoretical, we feel that they warranted an investigation and thus formed the overall purpose of the study. | Added citations of Murata et al. and Bernardi et al. in Lines 53 and 295. Expanded on the 3 clauses with the benefits of T-L coordination as well as breathing and oxygen during exercise in Lines 53-62. |
| Lines 76-77: I’m still unsatisfied with the reach of this statement. The reference (11) used is regarding children and not during exercise. What is “optimally efficient”? Again I suggest to contrast with the study of Bernardi, 2017 which is amongst the few that have specifically investigated T-L coordination during running in healthy individuals. | We thank the reviewer for bringing this to our attention. This statement was intended to highlight that everyone has room to breathe more efficiently in terms of T-L coordination (i.e. output a phase angle value closer to 0 degrees) but the statement lacked the proper citations to convey this. A citation to Bernardi et al. has been added to convey that anyone, whether exercising or resting, has a phase angle greater than 0 and therefore could be more efficient. | Expanded on this statement in Line 87 by adding a citation to the work of Bernardi et al., which now more properly conveys that everyone has room to be breathing more efficiently in terms of T-L coordination. |
| Why is visual feedback a suitable method for breathing instruction during running? I cannot see how this might translate into field running, where most runners participate. Other similar studies have utilized audio or haptic feedback (e.g., Valsted, 2017). Can you please address this limitation/discussion point and indicate why this context is important? | We thank the reviewer for this important point. We have added additional description of this limitation/discussion point. We do believe that given the lack of knowledge regarding whether and how much someone’s breathing patterns can be improved with biofeedback, providing visual feedback during treadmill running is an important step. | Lines 359-367 in the future work and limitations section address the importance of investigating this breathing feedback system to gain an initial understanding of it and future plans for this feedback to potentially be applied in other forms that are suitable for outdoor running. |
| Do you have values for phase angle at or just before exhaustion for each participant? I see a large limitation present if phase angles were averaged across the entire trial, but likely to change substantially from start to end. I would like to see some segmentation of these data *if* there is any difference between time points (e.g., run quartiles). | We agree – we had a similar concern about a substantial change from start to end in phase angle. Therefore, we had initially performed a check when doing our analysis. However, it did *not* actually change significantly. Since there was no significant difference, we had not included that in the manuscript initially, but now we have added this point in the first paragraph of the Results and the first paragraph of the Discussion. | The first half and second half phase angle values have been added to the results section in Lines 231-234. |
| Lines 268-274 address why this study utilized an incremental test to exhaustion. I don’t think 272-274 address this fully. Is breathing really a primary constraint/determinant at maximal exertion during such tests? Maybe T-L coordination is more of a factor in running (vs. e.g., cycling) because of the increased postural demands of the respiratory musculature? I am still missing the justification for this study design, especially since you did not collect spiroergometric data. It would be helpful to indicate why/when breathing indeed is a limiting factor upon maximal exercise performance. Please add this detail. | The question of whether breathing is a primary constraint during this maximal exertion exercise or not is an important one. Given that the original paragraph in question primarily addressed potential limitations of the study, it has been moved from the discussion section to the future work and limitations section. While the exact time/role that breathing would be the limiting factor is unknown in this study, ensuring that participants reached a true exhaustion point on both days allows us to confidently analyze the role that breathing played during the exercise as a whole. This point, along with the potential for constant-pace protocols to be more beneficial for analysis in the future, have been added. | Moved this paragraph from the discussion section to the future work and limitations section (Lines 329-344). Expanded on the points that this study allowed us to review the role that breathing played as a whole across the 2 days of exercise to exhaustion and that constant-pace protocols could be more beneficial in Lines 335-344. |
| Lines 305-306: again, increased oxygen uptake- is increasing VO2max really the aim of this breathing technique? Is that what you have tested with an incremental test (without spiro)? Measures of “breathing efficiency” are typically something more like VE/VCO2 or BR/VE- these should be mentioned here (or in lines 330-331). | We thank the reviewer for bringing this to our attention. Increasing VO2max was not a goal of this study nor the breathing technique in general, but rather an exploratory statement to convey that it could be useful to explore if T-L coordination increases VO2max in future studies, especially given the previously discussed findings on tidal volume and maximal oxygen uptake by Murata et al. This statement has been revised in the new version of the manuscript. | Expanded on the previous statement in the manuscript in Lines 310-316. This included adding a citation to Amann’s work on how respiratory system-related mechanisms can impair oxygen delivery to locomotor muscles, and a mention that analysis of VE/VCO2 would also be beneficial for future studies. |
