# Supplementary material for: The influence of receiving real-time visual feedback on breathing during treadmill running to exhaustion
Source: PLoS One. 2022 Jul 7;17(7):e0270335. doi: 10.1371/journal.pone.0270335 (PMC9262203; doi:10.1371/journal.pone.0270335)
Supplement: Attachment — Submitted filename: Reviewer comments 2.docx [file pone.0270335.s003.docx]

Thank you to the authors for these meaningful improvements in the manuscript and for the detailed replies. I still have some concerns and open questions.

- Lines 52-53: while the second clause is welcome detail, the third does not fully substantiate the basis for this study to use T-L coordination. What does T-L coordination have to do with oxygen perfusion into the bloodstream? I don’t see a clear connection here either theoretically or experimentally based on the references. Maybe the real justification (especially in exercise) has to do with respiratory muscle fatigue? Or perhaps expiratory flow limitation? Please revise (also 289-290).
- Lines 76-77: I’m still unsatisfied with the reach of this statement. The reference (11) used is regarding children and not during exercise. What is “optimally efficient”? Again I suggest to contrast with the study of Bernardi, 2017 which is amongst the few that have specifically investigated T-L coordination during running in healthy individuals.
- Why is visual feedback a suitable method for breathing instruction during running? I cannot see how this might translate into field running, where most runners participate. Other similar studies have utilized audio or haptic feedback (e.g., Valsted, 2017). Can you please address this limitation/discussion point and indicate why this context is important?
- Do you have values for phase angle at or just before exhaustion for each participant? I see a large limitation present if phase angles were averaged across the entire trial, but likely to change substantially from start to end. I would like to see some segmentation of these data *if* there is any difference between time points (e.g., run quartiles).
- Lines 268-274 address why this study utilized an incremental test to exhaustion. I don’t think 272-274 address this fully. Is breathing really a primary constraint/determinant at maximal exertion during such tests? Maybe T-L coordination is more of a factor in running (vs. e.g., cycling) because of the increased postural demands of the respiratory musculature? I am still missing the justification for this study design, especially since you did not collect spiroergometric data. It would be helpful to indicate why/when breathing indeed is a limiting factor upon maximal exercise performance. Please add this detail.
- Lines 305-306: again, increased oxygen uptake- is increasing VO2max really the aim of this breathing technique? Is that what you have tested with an incremental test (without spiro)? Measures of “breathing efficiency” are typically something more like VE/VCO2 or BR/VE- these should be mentioned here (or in lines 330-331).
